# Supplementary material for: ShcD Binds DOCK4, Promotes Ameboid Motility and Metastasis Dissemination, Predicting Poor Prognosis in Melanoma
Source: Cancers (Basel). 2020 Nov 13;12(11):3366. doi: 10.3390/cancers12113366 (PMC7696252; doi:10.3390/cancers12113366)

ShcD binds DOCK4, promotes ameboid motility and metastasis dissemination, predicting poor prognosis in melanoma.

Ewa Aladowicz, Letizia Granieri, Federica Marocchi, Simona Punzi, Giuseppina Giardina, Pier Francesco Ferrucci, Giovanni Mazzarol, Maria Capra, Giuseppe Viale, Stefano Confalonieri, Sara Gandini, Fiorenza Lotti, Luisa Lanfrancone.

Figure S1 : Evaluation of the impact of ShcD silencing on MM27 cells

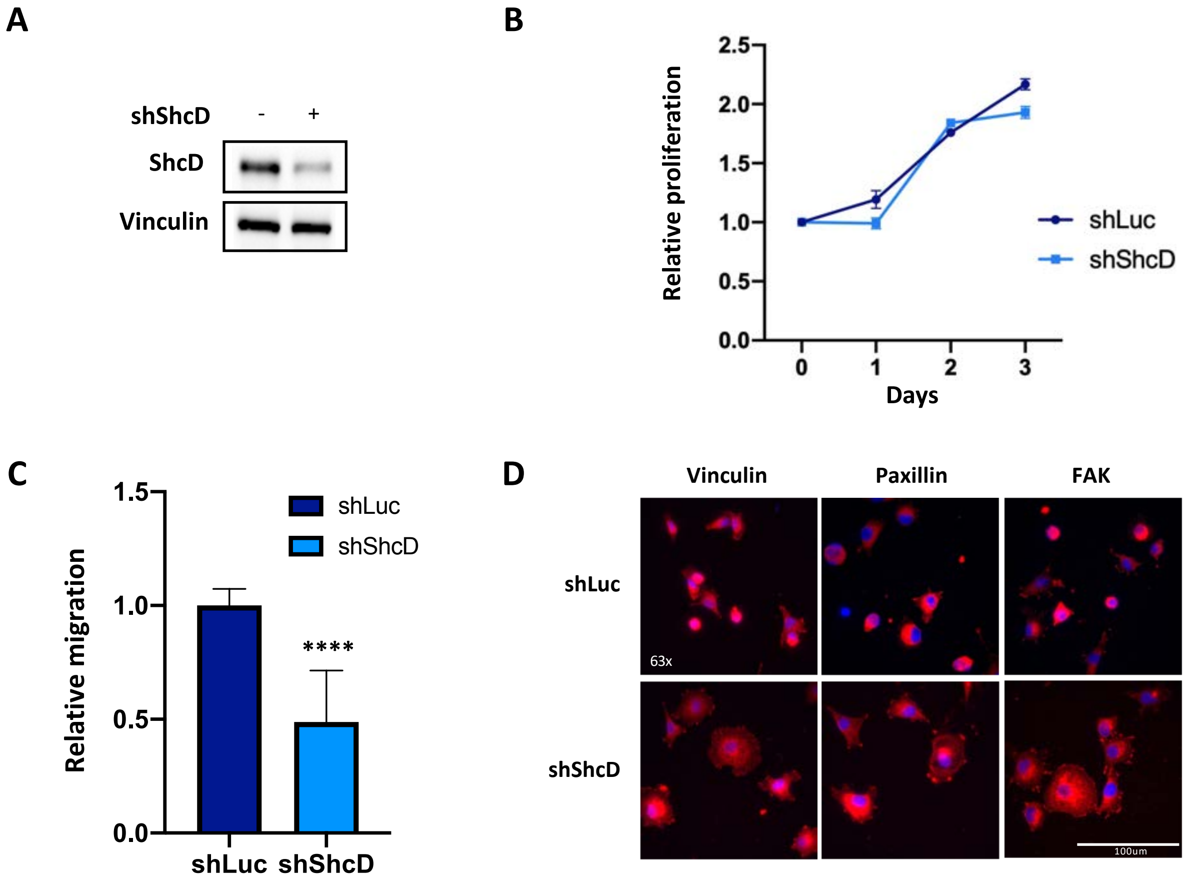

Figure S1. (**A**) Downregulation of ShcD in MM27 cells transduced with shShcD (pool of shShcD#1 and #2) or shLuc as a control was assessed by Western Blot analysis. Vinculin was used as a loading control. (**B**) Evaluation of the impact of ShcD silencing on cell proliferation. shLuc and shShcD (pool of shShcD#1 and #2) MM27 cells were plated for luminescent-based proliferation assay for 72h (Cell Titer-Glo, Promega). Proliferation values (mean ±SD) are expressed as ratio of the mean luminescent values in the shShcD and shLuc cells compared to the corresponding values at day 0. (**C**) Assessment of the impact of ShcD silencing on cell migration. Transwell migration was performed with shLuc and shShcD (pool of shShcD#1 and #2) MM27 cells at 48h time point. Relative migration (mean ±SD) is expressed as a ratio of silenced vs control cell migration values calculated by ImageJ analysis. Student *t-test* (****, P <0.001). (**D**) shLuc and shShcD (pool of shShcD#1 and #2) MM27 cells focal adhesion analysis by immunofluorescence. Cells were treated as in (Fig. 1B) and protein expression of vinculin, paxillin and FAK (red) was detected. Nuclei were counterstained with DAPI (blue). Representative images are shown (63x magnification).

Figure S2 : Cell morphology assessement in ShcD-silenced WM266.4 and WM115 cells

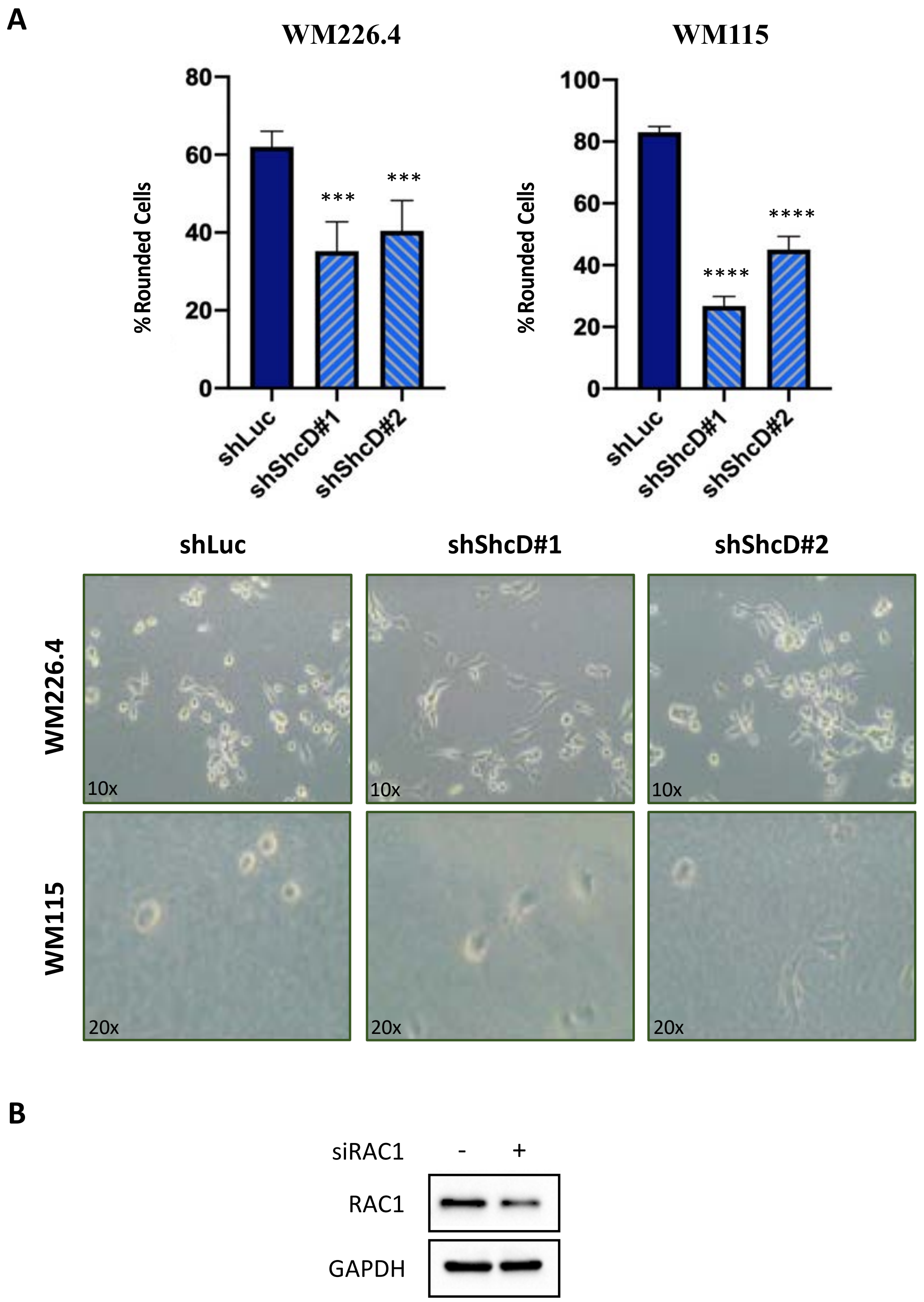

Figure S2. (A) shLuc control and shShcD#1, shShcD#2 WM266.4 and WM115 cells were plated on thick collagen layer and cell shape and percentage of rounded cells were calculated after 24h. Three images per well were acquired in triplicate experiments. Student t-test (***, *P* <0.01; ****, *P* < 0.0001). Representative images are shown. (**B**) WM115 cells were transfected with Rac1 siRNA SMARTpool. The silencing was quantified by Western Blot. GAPDH was used as house keeper.

Figure S3 : ShcD form a complex with DOCK4 and confines it in the cytoplasm

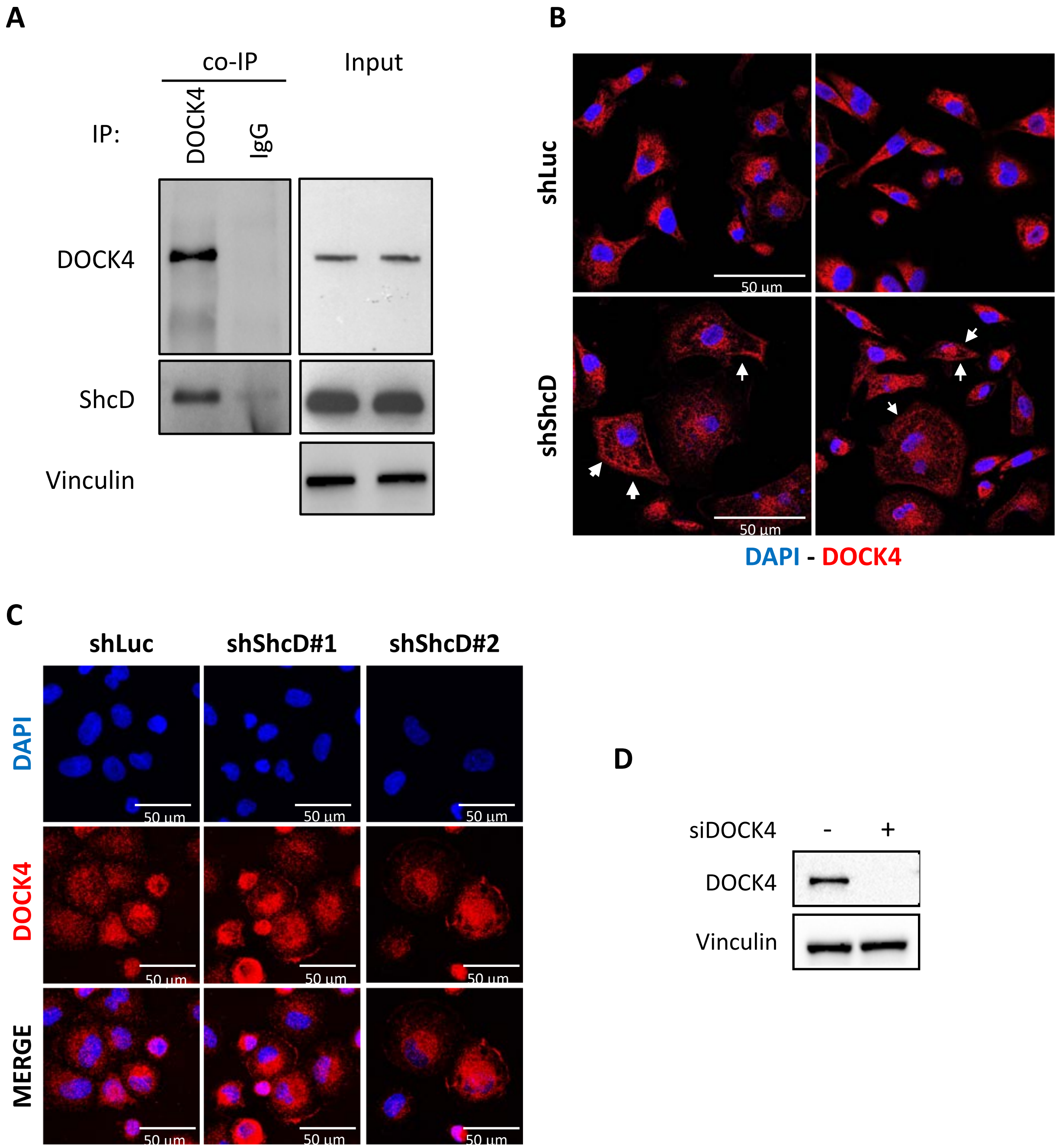

Figure S3. (A) DOCK4 and ShcD immunoprecipitation in WM115 cells. ShcD over-expressing WM115 cell lysates were immunoprecipitated (IP) with anti-DOCK4 antibody or anti-IgG antibody and subsequently immunoblotted with anti-ShcD antibody. Expression of vinculin was used as a loading control in total cell lysates (Input). **(B-C)** DOCK4 localization in MM27 (B) and shLuc, shShcD#1, shShcD#2 WM115 (C) cells by immunofluorescence analysis. MM27 and shLuc, shShcD WM115 cells were plated on fibronectin and stained for DOCK4 (red). Nuclei were counterstained with DAPI (blue). Representative images are shown (63x magnification). **(D)** MM27 cells were transfected with DOCK4 siRNA SMARTpool. The silencing was quantified by Western Blot. Vinculin was used as loading control.

All whole membranes from western blot images

Figure S1A

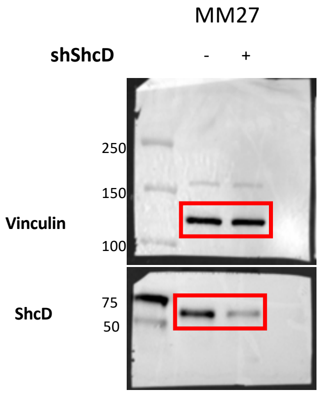

Figure 2A

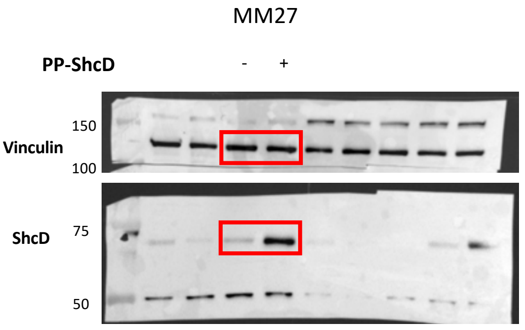

Figure 3 A

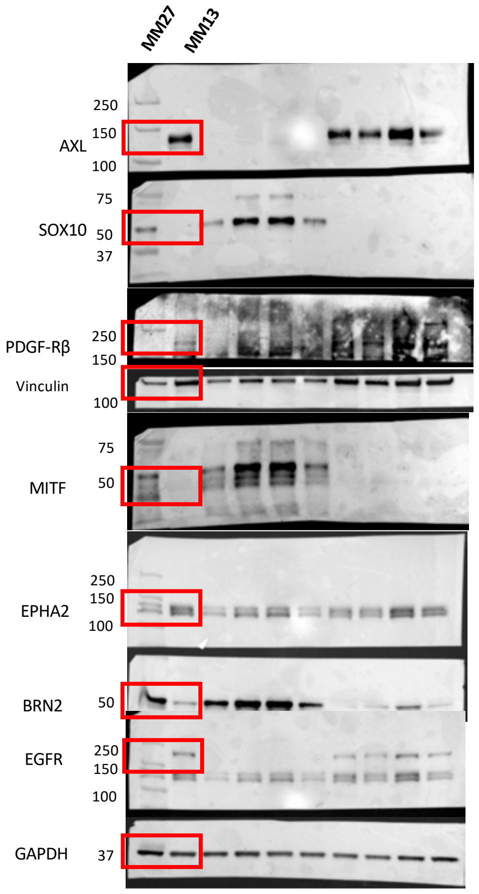

Figure 3 B

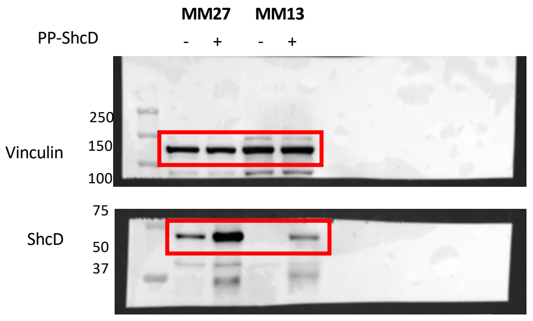

Figure 5C

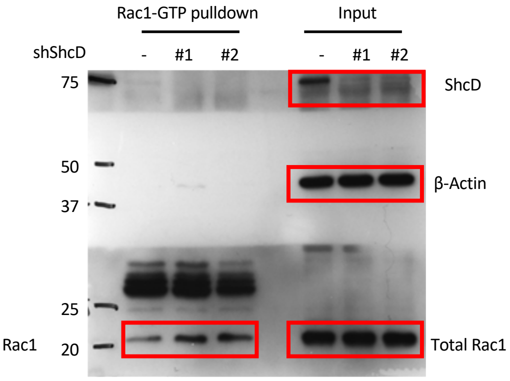

Figure 5D

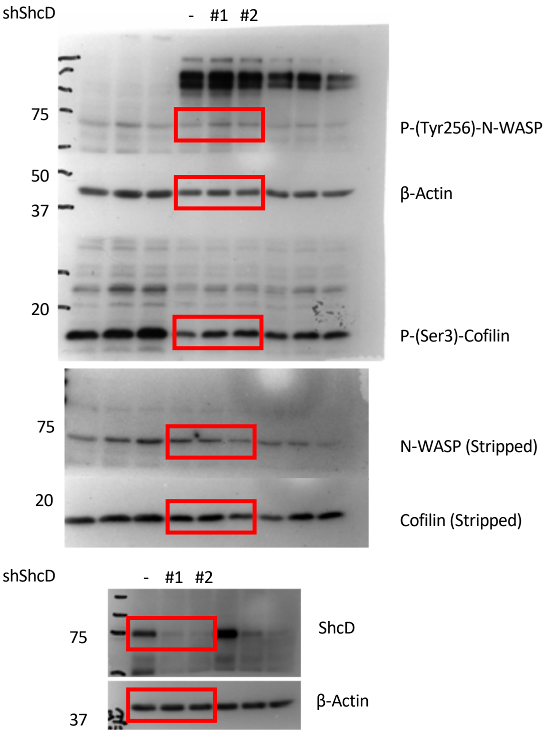

Figure 6A

Figure S3A

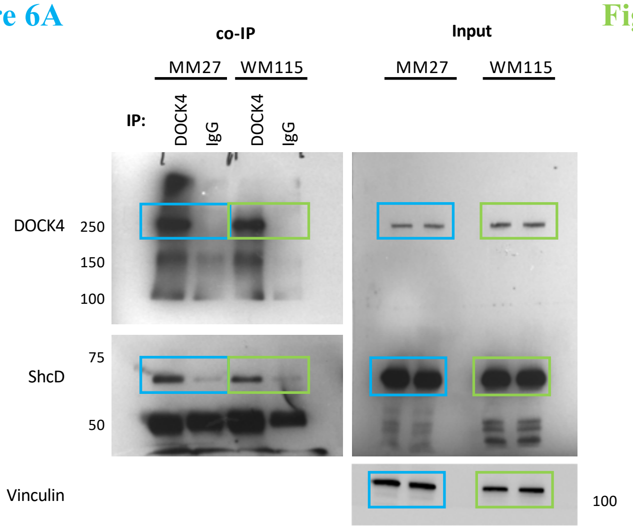

Figure S2B

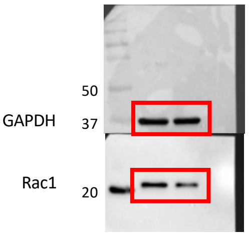

Figure S3D

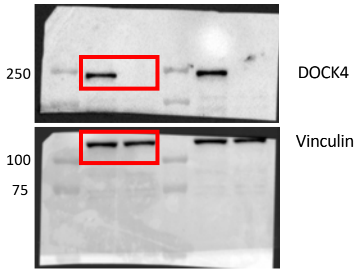

Supplement: Supplementary file 1 [file cancers-12-03366-s001.pdf]
